# Cannabinoid CB1 receptor-sensitive neurodevelopmental processes and trajectories

**DOI:** 10.1038/s41380-025-03057-2

**Published:** 2025-05-19

**Authors:** Kuei Y. Tseng, Hanna M. Molla

**Affiliations:** 1 Department of Anatomy & Cell Biology, University of Illinois at Chicago – College of Medicine, Chicago, IL USA; 2 Department of Psychiatry & Behavioral Neuroscience, University of Chicago, Chicago, Illinois USA

**Keywords:** Neuroscience, Addiction

## Abstract

As high-potency cannabis (with high Δ^9^-Tetrahydrocannabinol content) becomes easily accessible and widespread, it is of extreme importance for public health that a scientific platform is used to implement practical guidelines, particularly for at-risk populations. Many reviews have been written in the past decade summarizing the impact of cannabis in the developing brain. One critical concept frequently mentioned but not discussed in detail is whether there are sensitive neurodevelopmental events driving the age-specific sensitivity to cannabis, particularly those mediated by cannabinoid type 1 receptor signaling. By integrating available data from humans and animal models, the goal of the present expert review article is to provide a mechanistic overview on how cannabis exposure during sensitive periods of neural circuit plasticity and development can result in lasting consequences. Here we used the frontal cortex as a proxy to align the trajectory of the brain cannabinoid system between humans and rodents. Both the strengths and limitations of available mechanistic studies on the effects of cannabis and cannabinoids were discussed using a developmental framework from which neural circuit adaptations during sensitive periods are considered. Such an approach is needed to align key neurodevelopmental variables through the lifespan, which in turn will provide valuable insights applicable to the human brain by defining the underpinning mechanisms of sensitive periods and how the impact of cannabis changes from childhood to adolescence, and thereafter through young adulthood.

## Introduction

The terms cannabis and marijuana are often used interchangeably despite that they are technically distinct. While “cannabis” is used for all extracts derived from the cannabis plant, “marijuana” specifically refers to products made from *Cannabis sativa’s* or *Cannabis indica*’s dried leaves, flowers, stems and seeds (https://nida.nih.gov/research-topics/cannabis-marijuana) containing the psychoactive chemical Δ^9^-Tetrahydrocannabinol (Δ^9^-THC) [1]. According to the National Institute on Drug Abuse (NIDA), the **average** amount of Δ^9^-THC found in cannabis/marijuana seized by the DEA from 1995–2022 increased from 4–16% (https://nida.nih.gov/research/research-data-measures-resources/cannabis-potency-data). Currently, the amount of Δ^9^-THC found in marijuana can be as high as 40% or more, with low concentrations defined as <10%, and high concentrations as >15% (https://nida.nih.gov/research-topics/ cannabis-marijuana#cannabis-getting-stronger). In addition to Δ^9^-THC, there are more than one hundred cannabinoid constituents or phytocannabinoids that have been identified in the cannabis plant and classified into 11 types [2, 3]. Regardless of the term used to refer cannabis-derived products, a major challenge today is the application of sensitive research strategies to identify mechanisms by which the different types of cannabinoids impact neuronal activity and brain function through the lifespan.

According to the National Institute on Drug Abuse (NIDA) “Marihuana Research Findings: 1976” report, marijuana use among adolescents (12–17 years old) and young adults (18–25 years old) began to increase in the USA during the 1960’s. One of the main conclusions of the 1976 report stated that “*marihuana is most widely used by adolescents and young adults during sensitive stages in their personality development, while developing intellectual and psychosocial skills*”, and “*To what extent, if any, chronic intoxication affects development is still unknown*” (NIDA research monograph 14, 1976). This concern continues today as the maturation of executive functions such as working memory, decision making, and inhibitory control needed for the acquisition of adult behavior take place during the transitional period of adolescence [4–8]. Furthermore, there is converging evidence supporting the notion that early-onset cannabis use could increase the incidence of developing cognitive-associated psychiatric syndromes and substance use disorders later in life [9–17]. Certainly, establishing a causal link between cannabis use (e.g., frequency, age of onset, Δ^9^-THC content, route of administration) and the onset of mental illnesses remains a major challenge (see [18]). However, as high-potency cannabis (high Δ^9^-THC content) becomes easily accessible, it is extremely important for public health that well-designed mechanistic studies are used to establish a scientific platform for the implementation of practical policy and guidelines, especially for at-risk populations.

Numerous expert reviews and commentaries have been written in the past two decades on neurobiological consequences resulting from the developmental impact of Δ^9^-THC and the potential underlying mechanisms [19–30]. However, one critical concept that is often mentioned but not discussed in detail is the extent to which sensitive neurodevelopmental processes contribute to the age-specific vulnerability to Δ^9^-THC. Thus, the goal of the present review article is to provide a mechanistic overview focusing on cannabis-sensitive periods of neural circuit plasticity and development, particularly those processes that are sensitive to Δ^9^-THC and cannabinoid type 1 receptor (CB1R) signaling, which are linked to the detrimental impact of cannabis. We will use the frontal cortex as a proxy to integrate available data of the brain endocannabinoid system and match the most conserved developmental variables [31] to align the trajectories between humans and rodents. We will then elaborate and discuss the possibility that fluctuations in the trajectories of pre- and postsynaptic adaptations during the development and maturation of distinct neural circuits could dictate the length of a sensitive period and its end. Finally, we will highlight both the strengths and limitations of available mechanistic studies aimed at elucidating the neurobiology underlying the developmental impact of cannabis/cannabinoids by integrating the major findings obtained from rodents and humans.

## Developmental trajectories of the brain endocannabinoid system

It is well established that the different effects of Δ^9^-THC on neuronal function and behavior are mediated by the activation of the CB1R and associated postsynaptic signaling events [32–35] including those observed in human subjects [36–39]. In fact, treatment with the CB1R antagonist rimonabant can effectively attenuate major behavioral changes resulting from Δ^9^-THC exposure such as altered perception and distractibility, working memory deficits and increased anxiety and euphoria [36, 38]. As one of the most abundant G-protein coupled receptors in the brain [40–42], stimulation of CB1R with exogenous ligands is expected to alter the balance of excitatory and inhibitory synaptic activity [43, 44] and its control of neural circuit plasticity and behavioral responses [45, 46]. It is therefore conceivable that the neural mechanisms underlying the age-dependent susceptibility to cannabis are modulated by the developmental trajectory of the brain endocannabinoid system including the expression of CB1R.

**Fig. 1:**
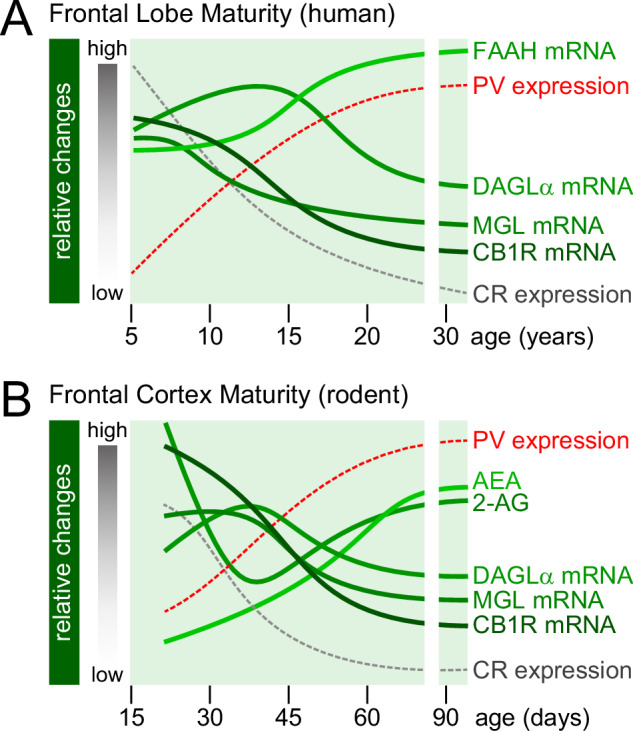
Comparative developmental trajectories of the brain endocannabinoid system: human vs. rodent frontal cortex [47–51]. **A** Summary of neurobiological variables measured in human postmortem studies. The patterns of parvalbumin (PV) and calretinin (CR) mRNA expression (Caballero and Tseng, [31]) were included to align the trajectories of the different endocannabinoid components observed in the human frontal cortex with those found in rodents [47–51]. **B** Using this developmental framework, a 5-day window in rats would be equivalent to 3 years in humans [31]. A major shift in the trajectories of the rodent frontal cortex endocannabinoid system emerges between postnatal days (P) 30 and 60 [47–51]. Note that the human frontal cortex experiences similar patterns of changes within the 10–20 years of age period. AEA N-arachidonoylethanolamine or anandamide, CB1R cannabinoid type-1 receptor, CR calretinin, DAGLα diacylglycerol lipase-α, MGL monoacylglycerol lipase, PV parvalbumin, 2-AG 2-arachidonoylglycerol.

A key challenge in developmental neuroscience is the integration of neurobiological variables across species with different lifespans. By using two conserved markers of cortical interneurons and the frontal cortex [31], we projected that a 5-day developmental window in rodents would be equivalent to 3 years in humans as estimated by the concurrent opposing changes of calretinin (CR) and parvalbumin (PV) expression between 12 and 20 years of age in humans and postnatal days (P) 35 and 55 in rats (Fig. 1). Through this approach and by integrating available data from the human and rodent frontal cortex, we were able to align the distinct trajectories of the brain endocannabinoid components from childhood to young adulthood and equivalent postnatal days in rats (Fig. 1A, B).

Specifically, CB1R expression in the frontal cortex is highest at the onset of adolescence and begins to decline thereafter through young adulthood in both humans [47, 48] and rodents [49]. A similar trajectory was observed with the level of diacylglycerol lipase-α (DAGLα) and monoacylglycerol lipase (MGL) [48, 50], which are the synthesizing and degrading enzymes for the CB1R endogenous ligand 2-arachidonoylglycerol (2-AG), respectively. Interestingly, the patterns of DAGLα and MGL do not match with the trajectory of 2-AG levels based on available data from the rodent prefrontal cortex (Fig. 1B). Contrary to the level of the other endocannabinoid anandamide (N-arachidonoylethanolamine or AEA) that steadily increases through adolescence onto young adulthood [51], 2-AG levels follow a non-linear pattern [51], which is characterized by a major drop from the onset of adolescence and a steady increase thereafter to match the trajectory of AEA (Fig. 1B). Finally, data from human samples [48] suggests that the trajectory for the fatty acid amide hydrolase (FAAH) enzyme, which breaks down AEA, is opposite to those of DAGLα and MGL (Fig. 1A).

Future studies are needed to fill the developmental gaps in rodents (e.g., FAAH mRNA) and humans (e.g., 2-AG and AEA levels) and expand the current analyses to other brain regions. However, it becomes clear from the integration of available data through the lifespan that the distinct trajectories within the brain endocannabinoid system are conserved between humans and rodents (Fig. 1). This approach is expected to provide valuable insights applicable to humans by defining the mechanisms of sensitive periods and how the impact of cannabis on CB1R signaling and neural circuit plasticity changes from childhood and adolescence to adulthood [19, 52].

## CB1 receptor-sensitive developmental periods and the maturation of corticolimbic circuits

The recruitment of the brain endocannabinoid system is typically activity-dependent and utilizes a CB1R-mediated retrograde signaling to limit the gain of GABA and glutamate transmission [43, 44, 53, 54]. Due to the prevalence of the CB1R throughout the brain [49, 55] and its role on synaptic plasticity [53], it is conceivable that the neurobiology contributing to the age-dependent effects of cannabis and Δ^9^-THC is mechanistically linked to neural processes underlying the development of sensitive periods and neural circuit maturation [19, 52].

Different forms of activity-dependent plasticity (e.g., Hebbian) and homeostatic adaptations are needed at both excitatory and inhibitory synapses during the formation and maturation of neural circuits which occurs through a series of developmental stages that are sensitive to environmental factors, often refer as “critical” periods (see [56]). Hence, exposure to Δ^9^-THC is expected to trigger distinct maladaptive trajectories of brain development depending on the sensitive period and the underlying mechanisms driving neural circuit maturation at that stage. This developmental framework is certainly applicable to other types of drugs and environmental stimuli (e.g., stressors) and to delineate potential mechanisms by which a sensitive period ends.

### Cannabis exposure during the pre/perinatal period

The endocannabinoid system through the CB1R signaling is known to play a critical role in shaping the formation of neural circuit connectivity by modulating key neurodevelopmental events such as neuronal migration and proliferation, axonal growth and long-range axonal patterning [57–60]. As the formation of neural circuits begins, it is not surprising that pre/perinatal exposure to cannabis/cannabinoids results in lasting developmental dysregulation of synaptic function [61–68] that ultimately could lead to aberrant behavioral responses later in life, often within the cognitive and affective domains (see review by [21]). However, it remains unclear if all neurodevelopmental processes occurring during the entire prenatal-perinatal period are equally sensitive to cannabis regardless of the stage of development [69] and if the frequency of exposure and Δ^9^-THC content matters in conferring such susceptibility.

In human studies, converging evidence indicates that prenatal cannabis exposure can result in developmental delays in infants during the first 2 years of life [70]. Another observation with pre/perinatal cannabis exposure is the development of mental health problems that become apparent in early adolescence [71]. These findings suggest that an early life impact by cannabis could trigger a series of synaptic and neuronal maladaptive responses that will not emerge/become apparent until adolescence when the maturation of prefrontal-corticolimbic circuits [52] to support cognitive abilities associated with adult behavior occurs [5, 72]. A protracted onset of behavioral phenotypes following early developmental insults is not unique to cannabis, but a common phenomenon often linked to altered prefrontal circuit maturation and its meso-corticolimbic functional connectivity [73]. Supporting this hypothesis are recent studies from rodent models showing lasting behavioral changes following pre/perinatal cannabis exposure that can be attributed to a developmental disruption of the meso-corticolimbic system [61, 63, 74–77]. In fact, the mesolimbic circuit becomes disinhibited following prenatal and early postnatal Δ^9^-THC exposure, a developmental dysregulation affecting primarily male offspring [61, 76, 77]. Both plasticity at GABA and glutamatergic synapses modulating mesolimbic dopamine neurons are thought to contribute to the sex-related disruption [61, 76, 77]. Yet, the precise mechanisms driving such maladaptive developmental dysregulation remains elusive. Nevertheless, these findings reveal that early developmental exposure to Δ^9^-THC can lead to exaggerated mesolimbic dopamine responses that could lead to the development of addiction-like phenotypes, particularly within the male population [76, 78] and during sensitive windows of brain maturation such as adolescence.

Several molecular and epigenetic changes have been extensively discussed in the context of potential mechanisms driving the negative impact of cannabis exposure during prenatal and perinatal periods (e.g., [21, 23]). Of particular interest are preclinical studies in rodents showing that prenatal Δ^9^-THC exposure alters mRNA expression of the opioid peptide, proenkephalin [66], and reduces dopamine D2 receptor gene expression within the mesolimbic circuit through histone modifications [79]. These epigenetic alterations could be mechanistically linked to the disinhibited mesolimbic circuit state found in offspring exposed to Δ^9^-THC during prenatal and early postnatal development [76, 78] and the development of addiction-like phenotypes later in life. However, only a few studies have included aspects of developmental trajectory in the experimental design to reveal age-sensitive maladaptive processes underlying the neurobiological effect of cannabis at early life [61–64]. Certainly, studies aimed at identifying the mechanisms driving the normal developmental trajectory of key neurobiological variables are crucial to gain insights on how distinct neural circuits adapt and become vulnerable to early life insults, and the extent to which maladaptive events last through the life span.

### Developmental trajectories of sensitive neurobiological processes during adolescence

**Fig. 2:**
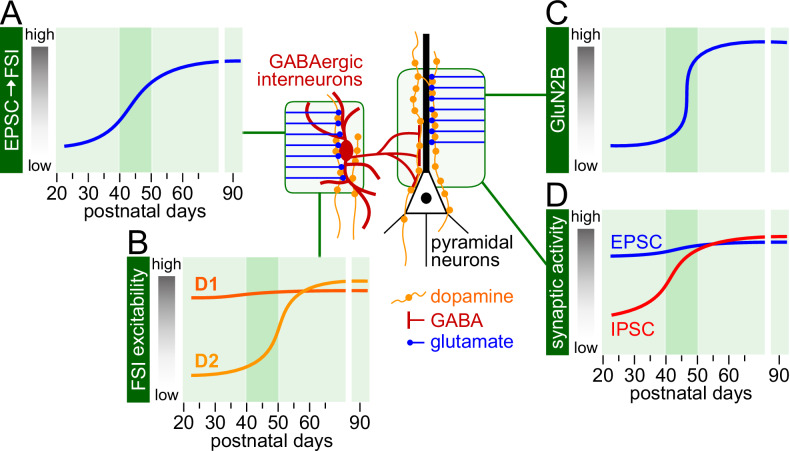
Developmental trajectories of prefrontal synaptic activity and dopamine modulation. **A** Trajectory of glutamatergic synaptic activity onto prefrontal cortical fast-spiking interneurons (FSI) as revealed by the frequency of excitatory postsynaptic currents (EPSC) [162]. **B** Trajectories of dopamine D1 and D2 receptors’ action onto FSI in the prefrontal cortex. Both D1 and D2 receptor stimulation resulted in facilitation of FSI excitability [163]. However, the D2 receptor effect begins to emerge after P45 [163, 164] through the acquisition of a non-canonical, β2-arrestin-mediated action [165]. **C** Trajectory of the GluN2B-mediated facilitation of synaptic activity in the prefrontal cortex, which occurs primarily onto the apical dendrite of layer V pyramidal neurons [81]. **D** Distinct developmental trajectories of spontaneous excitatory and inhibitory synaptic activity onto prefrontal layer V pyramidal neurons (see [93]). The frequency of inhibitory postsynaptic currents typically increases sharply after P40-45, a pattern that coincides with the increased EPSC frequency onto FSI shown in **A**. NOTE: The dark green shaded area highlights the adolescent period during which a shift in the trajectory of PFC glutamate and GABA synaptic function occurs.

It is well established that functional remodeling of key corticolimbic circuits continues through the sensitive period of adolescence [52] when the refinement of adult cognitive abilities such as problem solving, abstract thinking, and working memory takes place [5, 7, 72]. Such window of active remodeling creates a developmental period of sensitivity for environmental stimuli and insults to shape or alter the normal trajectories of neural circuit function (Fig. 2). Furthermore, the consumption of cannabis as well as the use of other drugs are expected to be amplified by the inherent novelty-seeking nature and increased risk-taking behaviors of the adolescent brain [6, 7]. Thus, a mechanistic analysis of the contributing factors driving and modulating the maturation of the corticolimbic circuitry is needed for understanding the neurobiology mediating the susceptibility to cannabis exposure during adolescence.

From the neurobiology standpoint, adolescence is currently viewed as a sensitive period during which major functional remodeling of local excitatory and inhibitory synaptic activity occurs in the prefrontal cortex following a unique age-related trajectory that directly impacts corticolimbic function and plasticity [52]. One key developmental factor shaping the maturation of corticolimbic circuits includes the functional recruitment of prefrontal GABAergic activity by excitatory afferents and dopamine during adolescence (Fig. 2A, B) that ultimately enables input selectivity and inhibitory control for sustaining working memory [80]. Such gain of synaptic activity in the prefrontal cortex during adolescence is certainly sensitive to cannabis exposure as the impact of CB1R signaling on prefrontal plasticity and function is age-dependent [45, 46, 49]. In addition to the gain of GABAergic output (Fig. 2A, B), is the strengthening of the ventral hippocampal-prefrontal pathway by the acquisition of GluN2B transmission during late adolescence (Fig. 2C) that also provides a mechanism for dopamine D1R modulation of input-specific afferent drive [80, 81]. Therefore, any disruption that compromises the normal gain of GluN2B and/or GABAergic function in the prefrontal cortex during adolescence (Fig. 2C, D) will alter the maturation of corticolimbic circuits and its impact on behavior.

Cannabis exposure during adolescence is expected to alter the trajectory of cognitive maturation [82] and its emotional regulation [83, 84] as these behavioral processes are mediated by significant changes in brain connectivity within the corticolimbic circuits [85, 86] expressing high levels of CB1R [47, 48]. As discussed above, the ventral hippocampal-prefrontal connectivity is of particular interest because it undergoes functional remodeling during adolescence [52] and is required for proper integration of contextual information [87–89]. Recently, a longitudinal study from human subjects (8–32 years of age) revealed that the enhanced hippocampal-prefrontal functional connectivity at rest [90] can predict the maturity of cognitive abilities to problem solving and future planning through adolescence [90, 91]. Such maturation of cognitive functions is thought to be mechanistically linked to neural processes underlying the re-calibration of the excitatory-inhibitory ratio that typically occurs in cortical circuits during adolescence both in humans [92] and rodents [93]. Notably, the underlying synaptic mechanisms driving the re-calibration of the excitatory-inhibitory balance in the prefrontal cortex are linked to the maturation of ventral hippocampal-prefrontal functional connectivity [94–98], which are all regions susceptible to transient disruption of CB1R signaling within discreet adolescent periods [45].

The formation of the corticolimbic system begins early in life and develops through childhood [56]. However, it is the gain of GABAergic function (Fig. 2A, B, D) together with the recruitment of GluN2B transmission in the prefrontal cortex (Fig. 2C) by ventral hippocampal inputs [81] that could explain the late adolescent maturation of cognitive and emotional regulation subserved by the different meso-corticolimbic circuits. Importantly, plasticity within these networks is also sensitive to the impact of CB1R signaling in an age-related manner [45, 46]. It is therefore crucial to distinguish the potential underlying mechanisms driving the distinct neural circuit susceptibility during adolescence compared to older age groups.

### Consequences of cannabis exposure during adolescence

Studies using rodent models have provided important evidence on how exposure to Δ^9^-THC or synthetic cannabinoids during adolescence, particularly before P50, affects neuronal and synaptic activity within the meso-corticolimbic system (Table 1). For instance, exposure to Δ^9^-THC within the P35-45 period led to inhibition of endocannabinoid-mediated long-term depression in the prefrontal cortex when assessed in adulthood [99, 100]. Δ^9^-THC exposure during a similar developmental window (P28-43) also reduced neuronal excitability in the prelimbic prefrontal cortex and attenuated NMDAR-mediated responses [101], which are essential for strengthening excitatory inputs from limbic structures such as the ventral hippocampus [81, 102]. Further, repeated exposure to the CB1R agonist WIN between P35-40 and P47-52 suppressed oscillatory activity in the prefrontal cortex [35], which are known to be developmentally regulated and dependent on local NMDAR transmission and postsynaptic D1R signaling [103, 104]. Similarly, Δ^9^-THC exposure between P35-45 markedly attenuated the activity of pyramidal neurons in the hippocampus causing a shift in theta and beta oscillatory power across both the dorsal and ventral hippocampus [105]. Together, these findings provide a mechanistic framework for understanding how repeated Δ^9^-THC exposure during adolescence compromises information processing within the hippocampal-prefrontal network and the resulting deficits in higher-order cognitive function in adulthood.

**Table 1:** Changes in neural activity and synaptic transmission following repeated cannabis/cannabinoid exposure during adolescence in rodents.

Changes in Neuronal and Synaptic Activity	Age	Drug	References
Inhibition of endocannabinoid-mediated LTD in the adult medial prefrontal cortex (layer 2/3)	P35-45	THC	Cuccurazzu et al. [99]
Lower firing rate and bursting activity of pyramidal neurons in the dorsal hippocampus; Higher theta power in the ventral hippocampus; Higher beta power in the dorsal hippocampus and lower beta power in the ventral hippocampus	P35-45	THC	De Felice et al. [105]
Reduced activity of dorsal raphe neurons in both aged groups.	P30-50, P50-70	THC	De Gregorio et al. [106]
Enhanced activity of dopamine neurons in the ventral tegmental area.	P25-58	THC	Kruse et al. [107]
Reduced excitability of prelimbic prefrontal cortex neurons; Attenuated depolarizing response of prelimbic prefrontal cortex neurons to NMDA.	P28-43	THC	Pickel et al. [101]
Reduced dopamine cell response to WIN in both groups.	P35-38, P56-59	WIN	Pistis et al. [108]
Suppression of oscillatory activities in the medial prefrontal cortex.	P35-40, P47-52	WIN	Raver and Keller [35]
Increased neuronal firing in the ventral tegmental area after adolescent exposure.	P35-45, P65-75	THC	Renard et al. [115]
Reduced neuronal firing in the dorsal raphe; Increased neuronal firing in the locus coeruleus.	P30-50	WIN	Rodriguez et al [62]
Impaired endocannabinoid-mediated LTD in the prefrontal cortex	P35-45	THC	Rubino et al. [100]
Decreased WIN-induced dopamine firing in the ventral tegmental area and reduced dopamine levels in nucleus accumbens.	P45-55	THC	Scherma et al. [109]
Frequency dependent disinhibition local field potentials in the prefrontal cortex; Reduced GABAergic transmission onto layer V pyramidal neurons of the prefrontal cortex.	P35-40, P40-45, P50-55, P75-80	WIN	Cass et al. [45]

*GABA* gamma-aminobutyric acid, *LTD* long-term depression, *NMDA* N-methyl-D-aspartate, *P* postnatal day.

Cannabis exposure during adolescence also impacts key neurotransmitter systems involved in mood and reward processing (Table 1). Δ^9^-THC exposure from P30-50 led to reduced activity of dorsal raphe neurons [106], which are part of the serotonergic system that plays a critical role in mood regulation. In addition, Δ^9^-THC exposure between P25-45 increased the activity of dopamine neurons in the ventral tegmental area, a region central to the brain’s reward circuitry [107]. On the other hand, an opposite effect on dopamine activity was found following cannabinoid exposure between P35-38 [108, 109], further indicating that age and/or duration of exposure matters when considering the long-term impact of cannabis and cannabinoids on neural function.

Table 2 summarizes how adolescent cannabis/cannabinoid exposure can trigger a wide range of biochemical and structural adaptations in the brain. One of the notable changes includes protein expression within various neurotransmitter systems. Δ^9^-THC exposure during adolescence downregulated dopamine receptor D2 (drd2) and adenosine A2A receptor (adora2a) gene expression in the nucleus accumbens and hippocampus, impairing dopaminergic and adenosine signaling pathways [110]. Exposure to Δ^9^-THC also altered the expression of receptors within both the glutamatergic and GABAergic transmission system. In the dorsal hippocampus, Δ^9^-THC exposure reduced the expression of NMDAR subunits, while in the ventral hippocampus, it increased these subunits and mGluR2/3 levels, contributing to region-specific alterations [105]. In the prefrontal cortex, Δ^9^-THC exposure during adolescence downregulates the levels of GAD65 in the prefrontal cortex, an enzyme critical for GABA synthesis [111], and disrupted the excitatory-inhibitory signaling balance by reducing GABA-Cnr1 expression and increasing glutamatergic Cnr1+ cells [11]. Both cortical GABA and glutamatergic systems are also sensitive to synthetic cannabinoids like JWH-018 and WIN [112, 113]. For instance, JWH-018 exposure reduced GAD67 expression in the prefrontal cortex and weakened local perineuronal nets, which are thought to contribute to the long-term neurobiological and behavioral deficits [112]. Similarly, WIN exposure through a self-administration protocol (i.v.) during adolescence altered the expression of proteins regulating GABAergic and glutamatergic signaling in the prefrontal cortex [113]. However, these changes resulting from WIN self-administration were not correlated with behavioral impairments, which is likely due to the different (lower) doses of WIN received during self-administration to those typically used in experimenter-administered studies (see [113]). Together, these findings are consistent with an earlier functional study showing that prefrontal inhibitory transmission is sensitive to disruption by repeated WIN exposure during adolescence [45]. In addition, the frequency-dependent disinhibition and the reduced GABA transmission observed in the prefrontal cortex following WIN exposure during adolescence were detected only when the treatment occurred prior to P50 [45]. Collectively, the data revealed that there are distinct windows of vulnerability through adolescence.

**Table 2:** Biochemical and structural changes in brain following repeated cannabis/cannabinoid exposure during adolescence in rodents.

Biochemical and Structural Changes	Age	Drug	Citation
Increased kynurenic acid levels in the adult prefrontal cortex.	P35-45	THC	Beggiato et al. [123]
Downregulation of drd2 and adora2a gene expression in the adult nucleus accumbens and hippocampus.	P35-57	THC	Cajiao-Manrique et al. [110]
Dorsal hippocampus: decreased GluN2B and GluN2A Ventral hippocampus: Increased GluN2B and mGluR2/3	P35-45	THC	De Felice et al. [105]
Increased AEA, reduced Met-enkephalin and µOR in the nucleus accumbens when tested during adolescence	P28-49	THC	Ellgren et al. [51]
Reduced GABA-Cnr1 expression and increased glutamatergic Cnr1+ cells in the prelimbic prefrontal cortex.	P28-59	THC	Ferland et al. [11]
Decreased expression of GAD67 and CB2R and perineuronal nets in the prefrontal cortex (prelimbic and infralimbic).	P35-49	JWH-018	Izquierdo-Luengo et al. [112]
^Increased GABA^B^R2, GAT, pNR2B in the prefrontal cortex; Reduced^ GAD65 in the prefrontal cortex.	P38-49	WIN	Kirschmann et al. [113]
Downregulation of CB1R expression on vGlut1 terminals in the ventral tegmental area.	P25-58	THC	Kruse et al. [107]
Premature dendritic spine pruning; altered functional gene networks.	P28-49	THC	Miller et al. [116]
Altered gene activation/deactivation in the nucleus accumbens.	P28-44	THC	Orihuel et al. [118]
Decreased GluN1-NMDA levels in the prelimbic prefrontal cortex.	P28-43	THC	Pickel et al. [101]
Increased histone modifications in the amygdala, hippocampus, nucleus accumbens in adolescent-exposed animals.	P35-45, P75-85	THC	Prini et al. [117]
Differential proteomic changes in the hippocampus.	P32-52, P64-84	THC	Quinn et al. [114]
Adolescent exposure: decreased phosphorylation of GSK-3α/β, Akt Thr308, mTOR, p70S6 Kinase, and β-Catenin in the prefrontal cortex. Adult exposure: increased phosphorylation of GSK-3α/β, Akt Ser473, and mTOR in the prefrontal cortex.	P35-45, P65-75	THC	Renard et al. [115]
Altered CB1R G-protein coupling in the amygdala, ventral tegmental area, nucleus accumbens; Altered CREB activity in the hippocampus, prefrontal cortex, and nucleus accumbens.	P35-45	THC	Rubino et al. [121]
Reduced CB1R binding, lower AEA and higher 2-AG; higher levels of GluN2B and GluA1.	P35-45	THC	Rubino et al. [100]
Reduced CB1R, GABA-AR, and glutamate receptor protein level in the prefrontal cortex, dorsal hippocampus, and ventral tegmental area.	P32-51	THC	Stringfield et al. [24]
Increased striatal levels of CB1R protein expression independent of sex. Changes in hippocampal CB1R levels varied by sex.	P35-75	THC	Weed et al. [119]
Reduced GAD67 in CCK- and PV-positive interneurons in the prefrontal cortex; Reduced basal levels of GABA.	P35-45	THC	Zamberletti et al. [111]
Increased pro-inflammatory markers in the prefrontal cortex: TNF- alpha, iNOS, COX-2; Reduced anti-inflammatory markers in the prefrontal cortex: IL-10.	P35-45	THC	Zamberletti et al. [122]

*2-AG* 2-arachidonoylglycerol, *adora2a* adenosine A2A receptor gene, *AEA* anandamide, *Akt Ser473* protein kinase B at serine 473, *Akt Thr308* protein kinase B at threonine 308, *Arc* activity-regulated cytoskeleton-associated protein, *β-Catenin* catenin beta-1 protein, *CB1R* cannabinoid type 1 receptor, *CB2R* cannabinoid type 2 receptor, *CCK* cholecystokinin, *Cnr1* cannabinoid receptor 1 [human], *COX-2* cyclooxygenase-2, *CREB* cyclic AMP-responsive element binding protein, *drd2* dopamine receptor D2 gene, *GABA* gamma-aminobutyric acid, *GABA-AR* gamma-aminobutyric acid type A receptor, *GABABR2* gamma-aminobutyric acid B receptor subunit R2, *GAD65* glutamic acid decarboxylase 65, *GAD67* glutamic acid decarboxylase 67, *GAT* gamma-aminobutyric acid transporter, GluA1 glutamate ionotropic receptor AMPA type subunit 1, *GluN1* glutamate ionotropic receptor NMDA type subunit 1, *GluN2A* glutamate receptor ionotropic NMDA 2A, *GluN2B* glutamate receptor ionotropic NMDA 2B, *GSK-3α/β* glycogen synthase kinase-3 alpha/beta, *IL-10* interleukin 10, *iNOS* inducible nitric oxide synthase, *Met-enkephalin* methionine-enkephalin, *mGluR2/3* metabotropic glutamate receptor 2/3, *mTOR* mammalian target of rapamycin, *NMDA* N-methyl-D-aspartate, *P* postnatal day, *p-CREB* phosphorylated cyclic AMP-responsive element binding protein, *pNR2B* phosphorylated N-methyl-D-aspartate receptor subtype 2B, *PV* parvalbumin, *TNF-alpha* Tumor necrosis factor alpa, *vGlut1* vesicular glutamate transporter 1, *µOR* mu-opioid receptor.

Adolescent cannabis/cannabinoid exposure also induces biochemical changes in key signaling pathways that regulate synaptic transmission and plasticity (Table 2). An early proteomic study [114] suggested that synaptic plasticity within the hippocampus is compromised after adolescent Δ^9^-THC treatment. In the prefrontal cortex, decreased phosphorylation of proteins such as GSK-3α/β, Akt, and mTOR were observed following adolescent Δ^9^-THC exposure, while an opposite effect was found when Δ^9^-THC was given in adulthood [115]. At the structural level, adolescent Δ^9^-THC exposure can lead to premature dendritic spine pruning and negatively impact the maturation of synaptic connectivity and plasticity [116]. Such structural changes are often linked to alterations in epigenetic adaptations and gene networks (Table 2), many of which are disrupted following Δ^9^-THC exposure during adolescence [117, 118].

As expected, the brain endocannabinoid system is also sensitive to long-term adolescent Δ^9^-THC exposure (Table 2). Major changes include anandamide and 2-AG levels [51, 100] and CB1R expression [51, 100, 107, 119, 120] and G-protein coupling [121] across different brain regions. Although several potential mechanisms may contribute to driving these alterations within the endocannabinoid system (see Section 2), it remains unclear whether the changes observed in CB1R expression and endocannabinoid levels following Δ^9^-THC exposure are strictly age-dependent. Finally, changes in pro- and anti-inflammatory markers were found in the prefrontal cortex following Δ^9^-THC exposure during adolescence [122], supporting the notion that non-neuronal signaling (e.g., astrocytes and glial cells) in the brain are likely to play a role in conferring the adolescent vulnerability to cannabis [123].

## Cannabis use and its impact on brain function in humans

As the access to cannabis is steadily rising [124], a major concern is the increased liability of developing neuropsychiatric syndromes such schizophrenia-spectrum disorders [9, 10, 13, 14, 17] and deficits in cognitive abilities later in life. Both short- and long-term effects on cortical function and its regulation of cognition are expected to emerge following cannabis exposure during adolescence [36, 43]. Unfortunately, studying the long-term impact of frequent cannabinoid use during adolescence presents several challenges, including the extended time frame required to assess long-term effects and difficulties accounting for key factors such as amount of cannabis intake, route of administration, frequency of use, and other environmental influences. Additionally, reliance on self-report data may introduce inaccuracies. Large-scale, long-term studies, such as the Adolescent Brain Cognitive Development (ABCD) Study, help to address these issues by collecting extensive data on over 10,000 children, over ten years. This allows researchers to track how cannabis use during adolescence impacts behavior and cognition into adulthood. While the study is ongoing, some data has already been examined (Table 3).

**Table 3:** Impact of chronic and acute cannabis exposure during adolescence in human subjects.

Human Studies	Age	Drug	References
**Chronic**			
Tested in adulthood and found impairments in reaction time of early users ( < 16 y/o).	12–16 y/o vs. >16 y/o	THC	Ehrenreich et al. [126]
Cannabis users exhibited riskier decision making and impulsivity in adulthood compared to non-frequent user age-matched controls.	Average age of initiation: 16.4 y/o	Used cannabis for a minimum of 25 days/month for 5 yrs	Ferland et al. [11]
Regular use ( > 1/week) led to poorer performance on attention and spatial working memory in adolescents who were tested.	13–18 y/o	Cannabis	Harvey et al. [12]
Cannabis use resulted in lower episodic memory scores than in healthy controls. More cannabis use was associated with worse performance on verbal, inhibitory, working and episodic memory tasks.	13–14 y/o	Cannabis	Wade et al. [125]
Higher cannabis use was associated with worse decision making and episodic memory one year later.	14–17 y/o	Cannabis	Duperrouzel et al. [127]
Cannabis users in both age groups showed poorer verbal episodic memory. No effects were observed in spatial working memory or response inhibition.	16–17 y/o vs. 26–29 y/o	Cannabis	Lawn et al. [128]
Cannabis users showed poorer verbal learning, working memory, and attention. Deficits in attention accuracy remained apparent after weeks of abstinence.	15–19 y/o	Cannabis	Hanson et al. [129]
**Acute**			
No differences between adolescents and adults on psychotomimetic effects, verbal memory, or subjective effects. No differences in effect of CBD.	16–17 y/o vs. 26–29 y/o	THC and THC + CBD	Lawn et al. [128]
Both age groups showed THC-induced reductions in network-connectivity in default mode within hippocampal and limbic striatal networks.	16–17 y/o vs. 26–29 y/o	THC and THC + CBD	Ertl et al. [130]
Adolescents showed impaired response inhibition and satiety, adults had more cognitive impairments in memory.	16–17 y/o vs. 24–28 y/o	Cannabis vaporized (0.89 mg/kg)	Mokrysz et al. [133]
Adolescents showed more impairments in reaction time, response accuracy, and time perception. EEG data revealed a reduction in P300 amplitude in a THC dose-dependent manner.	18–20 y/o vs. 30–40 y/o	THC oral (7.5 mg and 15 mg)	Murray et al. [132]

*EEG* electroencephalography, *P300* component of EEG event related potential, *P* postnatal day.

Analysis of a subset of 13–14-year-olds in the ABCD study found that those who self-reported cannabis users in the past year, and/or had cannabinoids detected in hair samples, had more impaired episodic memory than demographically matched controls [125]. Higher usage was associated with worse performance on tasks involving verbal, inhibitory, and memory related tasks, similar to findings from other studies [11, 12, 126, 127]. Other studies have shown that cannabis use affects cognitive processes like verbal episodic memory to a similar degree in both adolescents (16–17 years old) and adults (26–29 years old) [128], and these deficits could be reversed after 2–3 weeks of abstinence [129]. Data from these studies raises important questions about the specificity and reversibility of these effects, and more studies are needed to explore this further.

To explore differences in cannabinoid responses across developmental stages, researchers can examine the acute effects of Δ^9^-THC under controlled conditions, at different points during adolescence, as well as in later stages of maturation. This approach allows for control over important factors (e.g., dose, recency of exposure) while assessing differences in sensitivity across age groups. The few studies that have conducted such experiments have shown mixed findings. While some research has shown no differences in neural, cognitive, or subjective effects of Δ^9^-THC between adolescents (16–17 years old) and adults (26–29 years old) [128, 130], other studies suggest distinct age-related responses [131, 132]. For example, one study found that adolescents experienced greater impairments in response inhibition and satiety, while adults showed more memory-related impairments following acute cannabis administration [133]. Another study demonstrated that adolescents/young adults (18–20 years old) exhibited greater sensitivity to acute Δ^9^-THC, showing impairments in response inhibition, time perception, and altered neural activity measured by EEG during an attention task, which were not observed in adults (30–40 years old) [132]. These results suggest that certain cognitive and neural processes may be more vulnerable to acute Δ^9^-THC exposure during adolescence, while others may be less affected by age. Further studies are needed to better understand the nuances of age-related sensitivity to Δ^9^-THC.

A major detrimental impact resulting from chronic cannabis use is the development of cognitive deficits within the working memory and decision-making domains [134–137]. Data from rodent models of chronic cannabinoid exposure (see Section 3.3) indicate that sustained CB1 receptor stimulation could account for the onset of cognitive deficits observed [19]. While the level of CB1 receptor availability was not correlated with the duration or the age of onset of cannabis use [138], current available data shows that the degree of cognitive deficits observed in chronic cannabis users is linked with the extent of CB1 receptor stimulation in a dose-dependent manner [138, 139]. Accordingly, the density of CB1 receptors begins to recover within days of abstinence [140] reaching control levels after one month of abstinence [139], which coincides with the recovery of cognitive function [141, 142]. Similarly, the impact of chronic cannabis use during adolescence have been well studied over the years, with increasing research pointing toward negative impacts on prefrontal cortical-dependent cognitive processes (see [22]). For example, cannabis users who begin prior to age 16 develop attentional deficits that are not apparent in late onset users [126]. This observation is consistent with data obtained from regular cannabis users aged 13–18 exhibiting poorer performance in cognitive tasks within the attention and spatial working memory domains [12]. Long-lasting cognitive dysregulation observed in regular cannabis users within the adolescent population include attention deficits and impairments in executive function [135, 143]. However, future studies are needed to establish a causal link by assessing the extent to which genetic factors and childhood environments contribute to the lasting negative impact of regular cannabis use within the adolescent population (see [22]). As discussed above, there are several additional variables that need to be accounted for such as the amount, route, and frequency of cannabis intake.

## Practical considerations and concluding remarks

Extensive studies in both humans and rodent models have been conducted over the years to reveal the impact of repeated cannabis exposure during sensitive periods of brain development and maturation. Collectively, the data all point towards a detrimental effect on a series of cognitive processes that are reliant on the prefrontal cortex and its functional connectivity within the corticolimbic network [16, 19, 26, 131]. As mentioned in Section 1, many reviews have been written in the past two decades highlighting the long list of behavioral changes resulting from repeated cannabis and cannabinoid exposure during different stages of brain development [19–30]. For instance, adolescents are typically more sensitive to the effects of Δ^9^-THC and cannabinoids than adults both in rodents [45, 114, 144–149] and humans [132, 133, 150], which coincide with the higher level of CB1R expression in the brain of adolescents relative to adults (Fig. 1). Certainly, a developmental framework is needed to enable the distinction of age-dependent vs. age-independent effects of Δ^9^-THC and to uncover the underlying mechanisms driving the end of a sensitive period. However, it is also crucial to incorporate mechanistic aspects of the brain endocannabinoid system and how disruption of tonic versus phasic endocannabinoid signaling by Δ^9^-THC exposure contributes to alter synaptic plasticity [151–156]. Data from a recent study further suggest that the impact of Δ^9^-THC on CB1R function could be linked to the proximity to the effectors regardless of its expression level [157]. Using an in vivo pharmacological treatment paradigm that induces behavioral tolerance, it was found that the dose-dependent reduction of CB1R content in axonal boutons observed following THC exposure in adolescent mice has little functional consequences on phasic endocannabinoid synaptic transmission [157]. Instead, adolescent THC exposure decreased the intra/peri-synaptic CB1R population and the nanoscale receptor-to-effector ratio, which disrupts the nanoscale functional organization required for sustaining normal synaptic cannabinoid tone [157]. In this regard, establishing how nanodomain-specific processes are mechanistically linked to phasic vs. tonic control of synaptic function [157] and its impact on synaptic plasticity could provide clues on how Δ^9^-THC exposure affects neural circuit function during development [158–161]. Together, such knowledge will provide unique insights on why a developmental window is particularly vulnerable to environmental influences like cannabis and Δ^9^-THC, and whether the complex landscape of genes-environment interaction contributes to the underpinnings of individual differences. A mechanistic developmental analysis of cannabis/cannabinoid-sensitive neural circuits will delineate a much-needed research paradigm to uncover the distinct neurobiological effects of Δ^9^-THC through the lifespan.
